# Early Prediction of Hypertensive Diseases of Pregnancy by Using Combined Screening Methods in a Rural Population

**DOI:** 10.7759/cureus.50624

**Published:** 2023-12-16

**Authors:** Ruhida Razzak, Poonam V Shivkumar

**Affiliations:** 1 Obstetrics and Gynecology, Mahatma Gandhi Institute of Medical Sciences, Wardha, IND

**Keywords:** predictive model, mean arterial pressure, rural population, papp a, doppler ultrasonography, gestational hypertension pregnancy

## Abstract

Introduction: The most frequent medical issue during pregnancy is hypertension, which can complicate up to 10% to 15% of pregnancies worldwide. An estimated 14% of all maternal fatalities worldwide are thought to be caused by hypertensive disease of pregnancy, one of the main causes of maternal and fetal morbidity and mortality. Despite the fact that maternal mortality is substantially lower in high-income countries than in low- and middle-income countries, hypertension is still one of the leading causes of maternal death globally. Maternal mortality associated with hypertension fluctuated between 0.08 and 0.42 per 100,000 births between 2009 and 2015. In India, the estimated overall pooled prevalence of HDP was determined to be one out of 11 women, or 11% (95% CI, 5%-17%). Despite various government programs, there is still a high prevalence of hypertension, which calls for stakeholders and healthcare professionals to focus on providing both therapeutic and preventive care. The best solution is to concentrate more on the early detection of pregnancy-related hypertension and to guarantee its universal application so that proper care can be carried out to prevent maternal and fetal morbidity.

Aim: To estimate the predictive value of the combination of maternal characteristics, i.e., mean arterial pressure (MAP), biophysical evaluation (uterine artery Doppler), and biochemical markers (pregnancy-associated plasma protein A (PAPP-A)), in the first trimester of pregnancy for hypertensive diseases of pregnancy.

Methodology: It was a prospective observational study of longitudinal variety that took over 18 months in a tertiary care rural hospital. The number of women admitted to the hospital for labor care during 2019 was 5261. A total of 513 were diagnosed with hypertensive illnesses during pregnancy. At a prevalence rate of 10%, we calculated a sample size of 350 to achieve a sensitivity of 85% with an absolute error of 12.5% at a 95% CI. Maternal histories, such as age, education, socio-economic status, gravidity, and BMI, were taken along with three parameters, i.e., MAP, which was significant above 90 mmHg, uterine artery Doppler, which was taken significant above 1.69, and serum PAPP-A, which was significant at less than 0.69 ml/IU.

Observation and results: We have found that the following are associated with the prediction of hypertension: among the maternal characteristics are advanced age >35 years, presence of body edema, and urine proteins along with MAP, uterine artery pulsatility index (UtA-PI), and PAPP-A are significant. The predictive accuracy of the combination of MAP, UtA-PI, and PAPP-A is also significant. We also found that there is a significant increase in cesarean sections and NICU admissions in hypertensive patients.

Conclusion: A combination of screening parameters, including MAP, UtA-PI, and PAPP-A, to predict early hypertensive disease of pregnancy is developed and tested.

## Introduction

Hypertension, the most common medical problem during pregnancy, may complicate as many as 10-15% of pregnancies throughout the globe. Hypertensive disease of pregnancy (HDP) is a leading cause of maternal and fetal morbidity and mortality, accounting for an estimated 14% of all maternal deaths globally. HDP remains among the major causes of maternal mortality worldwide, even though maternal mortality is far lower in high-income countries (HICs) than in low- and middle-income countries (LMICs).

Between 2009 and 2015, the rate of HDP-related maternal fatalities ranged from 0.08 to 0.42 per 100,000 live births, with a percentage of 2.8% in the United Kingdom and Ireland (2011-2013). Projections show that HDP causes 7.4% of maternal fatalities in the United States and accounts for one-fifth of prenatal hospitalizations and two-thirds of referrals to day assessment facilities. In France, HDP accounts for one-fourth of all obstetric ICU hospitalizations. However, in LMICs, HDP is associated with 10-15% of direct maternal mortality [1]. It is estimated that 116.4 of every 100,000 women of childbearing age live with HDP. The highest regional mean HDP prevalence was in Southeast Asia (136.8 per 100,000 women of reproductive age) and the Middle East (121.4 per 100,000 women of childbearing age). With a mean frequency of 334.9 per 100,000 reproductive-age women in Africa, the continent had the highest overall HDP incidence. The Western Pacific region had the lowest HDP frequency, with 16.4 cases per 100,000 women of childbearing age. When comparing HICs and LMICs, there is a significant gap in the HDP illness burden [1]. Inequity in the average prevalence of HDP is seen worldwide. The average HDP incidence is highest in Africa compared to any other continent. Next, HDP has a mean prevalence of above 0.1% among women of reproductive age in the Eastern Mediterranean and South East Asia. In the Western Pacific, the average rate of HDP is the lowest [1].

In India, there are regional variations in the prevalence of hypertensive diseases during pregnancy. It is estimated that the overall pooled prevalence of HDP was one out of 11 women, or 11% (95% CI, 5%-17%). Despite various government programs, there is still a high prevalence of hypertension, which calls for stakeholders and healthcare professionals to focus on providing therapeutic and preventive care. The best solution is to concentrate more on the early detection of pregnancy-related hypertension and to guarantee its universal application so that proper care can be carried out at the proper time and location to reduce maternal and fetal morbidity and mortality [2].

Classifying hypertensive illnesses in pregnancy into four categories is the recommendation made by the National High Blood Pressure Education Program Working Group on High Blood Pressure in Pregnancy: (1) gestational hypertension (transient hypertension of pregnancy included); since this word is more specific, it has replaced the more generic “pregnancy-induced hypertension” (PIH); (2) preeclampsia-eclampsia; (3) preeclampsia (PE) superimposed on chronic hypertension; (4) chronic hypertension [3,4].

PE is the most frequent hypertension disorder during pregnancy, and it may have devastating effects on the expectant mother and the unborn child. This subject warrants much investigation. After 20 weeks of pregnancy, hypertension and proteinuria signal the development of PE and HDP. PE affects 2-8% of pregnancies, yet it is a significant source of neonatal and maternal morbidity and death. Pregnancy leads to temporary physiological adaptations that can have extensive effects [5]. Despite extensive interest in the disease and its impact on maternal and fetal health, no effective treatment other than delivery of the placenta has been developed. Recent evidence suggests that PE can be further subdivided into early and late PE, the former being associated with a higher incidence of fetal growth restriction and both short and long-term maternal mortality and morbidity. According to an update published in 2014 by the International Society for the Study of Hypertension in Pregnancy (ISSHP), PE is defined as the de novo emergence of hypertension after the 20th week of pregnancy in addition to signs of maternal organ failure, which include the following: new-onset proteinuria of greater than 300 mg per day or other signs of renal insufficiency, hematological issues like thrombocytopenia and liver dysfunction, neurological issues like visual disturbance, and/or signs of uteroplacental issues like fetal growth restriction [6].

It is thought that in women with PE, a complex interaction between placental factors, maternal constitutional factors, and pregnancy-specific vascular and immunological adaptation occurs in the first trimester of their pregnancy. The clinical manifestations of PE, such as high blood pressure and proteinuria, are only terminal features of this cascade of events. Therefore, early recognition of women at risk and timely intervention ahead of clinical onset might enable tailored pregnancy care and better pregnancy outcomes. A multisystem pregnancy disease, PE is characterized by varying degrees of placental malperfusion and the release of soluble substances into the bloodstream. These elements harm the vascular endothelium of the mother, which results in hypertension and organ damage. Fetal growth limitation and stillbirth can result from placental illness [7,8]. By contrast, late-onset PE is linked to milder maternal illness and a lower risk of fetal involvement, with delivery occurring at or after 34 weeks. Late-onset PE often results in not discouraging perinatal outcomes. After issues have been identified, clinical care and proper monitoring may begin sooner if PE is detected early. Prophylactic treatments for PE starting in mid-pregnancy are not effective in clinical trials. The purpose of introducing specific measures for better maternal and infant health is to reduce and improve maternal-fetal outcomes [8,9].

A reliable diagnostic test for this condition is crucial for reducing death rates. To date, no one test has shown enough predictive value for PE to be used in clinical practice. These tests are most helpful when used in conjunction with other variables. Due to the diverse character of PE, it may be easier to build appropriate prediction algorithms if many independent biomarkers are used. Multiparametric techniques, which take into account a large number of variables all at once, are the most successful in the prediction of PE. Pregnancies at high risk for early-onset PE may be identified by maternal risk indicators, such as uterine artery pulsatility index (UtA-PI), mean arterial pressure (MAP), and maternal serum pregnancy-associated plasma protein-A (PAPP-A) [10,11].

According to preliminary data from the National Eclampsia Registry (NER) of the Federation of Obstetric Societies of the United States and the International Confederation of Obstetricians and Gynaecologists, both HDP and eclampsia are on the rise, especially among cases handled in less affluent settings by medical personnel who lack appropriate education and experience. PE was reported to have a 10.3% incidence rate (NER 2013). More than half of all instances of eclampsia occur during pregnancy, and another 13% occur shortly after delivery. Eclampsia is responsible for a 4-6% fatality rate in pregnant women. There is an unmet need in LMICs for the recognition and management of HDP and its complications because of myths and misconceptions about pregnancy, difficulties in transportation facilities, low socioeconomic status, the need for a multidisciplinary approach, a lack of accurate prediction methods, and a scarcity of high dependency units (HDU) [12].

Hypertension in pregnancy is best managed by a multidisciplinary team that includes obstetricians, maternal-fetal medicine specialists, neonatologists, nephrologists, hypertension specialists, cardiologists, anesthesiologists, pharmacists, nurses, and midwives, all of whom work together to ensure the best possible outcomes for both mother and child before, during, and after pregnancy. When it comes to improving health outcomes, preventive approaches such as group prenatal care, evaluations of economic vulnerability and chronic stress, medication modifications, dietary advice, lifestyle counseling, and educational materials have been demonstrated to be effective [13]. By using early warning scores, hypertension bundles, and toolkits, nurses may identify maternal compromise sooner upon hospital admission, which has been linked to a decrease in maternal mortality due to hypertensive conditions [14]. Since PE is now recognized as a distinct risk factor for cardiovascular disease (CVD) by the American Heart Association, it has been included in the algorithms used to determine a woman’s future cardiovascular risk score [15-17]. PE, which raises blood pressure independently or in addition to chronic vascular illness, complicates around 5-7% of all pregnancies and is one of the primary causes of maternal and fetal morbidity. While premature birth is linked with acute neonatal morbidity, PE is an early predictor for the development of cardiovascular and other metabolic problems in the future [18,19]. Hypertensive diseases during pregnancy continue to be one of the least-researched and least-funded areas, as measured by disability-adjusted life years (DALYs). In turn, this leads to debates over how to best categorize, diagnose, and treat hypertension problems in pregnant women. Gestational hypertension and PE are the most prevalent forms of pregnancy-related hypertension.

Glossary of hypertension in pregnancy-related terms

Gestational Hypertension

Systolic blood pressure (SBP) ≥140 mmHg and/or diastolic blood pressure (DBP) ≥90 mmHg on at least two occasions at least four hours apart after 20 weeks of gestation in a previously normotensive patient without proteinuria or evidence of end-organ damage [20].

Pre-eclampsia

SBP ≥140 mmHg and/or DBP ≥90 mmHg on at least two occasions at least four hours apart after 20 weeks of gestation in a previously normotensive patient and with or without proteinuria and or evidence of end-organ damage. Proteinuria ≥0.3 g in a 24-hour urine specimen or protein/creatinine ratio ≥0.3 in a random urine specimen or dipstick ≥2+; platelet count <100,000/microL; serum creatinine >1.1 mg/dL or doubling of the creatinine concentration; rise in liver transaminases at least twice the upper limit of the normal; pulmonary edema; persistent headache; visual disturbances [20].

Preeclampsia With Severe Features

PE with severe features is considered if a patient with PE exhibits any of the following symptoms: SBP ≥160 mmHg and/or DBP ≥110 mmHg on two occasions at least four hours apart while the patient is on bed rest. New-onset cerebral or visual disturbance, such as photopsia, scotomata, cortical blindness, retinal vasospasm, and/or severe headache. Raised serum transaminase > two times the upper limit of the normal range and/or severe persistent right upper quadrant or epigastric pain. Serum creatinine >1.1 mg/dL or doubling of the creatinine concentration and platelet count <100,000/microL.

Eclampsia

When alternative causes of a generalized tonic-clonic seizure in a pregnant woman with PE have been eliminated, the diagnosis is PE.

Chronic (Pre-existing) Hypertension

Hypertension diagnosed or present before pregnancy with SBP >140 mmHg and or DBP >90 mmHg on at least two occasions before 20 weeks of gestation taken four hours apart.

Chronic Hypertension With Superimposed Preeclampsia

Any of these findings in a patient with chronic hypertension: a sudden increase in blood pressure that was previously well-controlled or an escalation of antihypertensive therapy to control blood pressure; new onset of proteinuria or a sudden increase in proteinuria in a patient with known proteinuria before or early in pregnancy; significant new end-organ dysfunction consistent with PE after 20 weeks of gestation or postpartum [20-22].

Pathophysiology

Pathophysiological explanations for PE may be found in interactions between the mother, the developing baby, and the placenta. Hypertension and other manifestations of the disease, including hematologic, cardiac, pulmonary, renal, and hepatic dysfunction, may arise from abnormalities related to the development of the placental vasculature early in pregnancy, leading to relative placental underperfusion/hypoxia/ischemia, which may release antiangiogenic factors into the maternal circulation [23].

Abnormal development of the placenta

The placenta plays an important part in the pathophysiology of PE. The fetus is not required for the development of PE, but the placental tissue is [24-26]. PE usually goes away within a few days to a few weeks after the placenta is delivered. Postpartum hypertension and PE have been reported to develop as late as eight weeks after birth. Some of the reasons that may contribute to this phenomenon include post-delivery complement activation, delayed clearance of antiangiogenic agents, and/or the mobilization of extracellular fluid into the intravascular compartment [27].

Researchers have studied human placentas at different stages of pregnancy, gaining insight into normal uteroplacental circulation that is potentially important to PE. Hypertensive diseases during pregnancy, as well as fetal growth limitation, have been linked to problems in spiral artery remodeling and trophoblast invasion [28].

Abnormal remodeling of spiral arteries

Blood supply to the developing baby and placenta typically originates from the maternal spiral arteries, the terminal branches of the uterine artery, via the invasion of placental cytotrophoblast cells through the endothelium and the highly muscular tunica media. The placenta receives blood via arteries that change from microscopic muscle arterioles into high-capacitance channels with low resistance [29]. Remodeling of the spiral arteries begins in the late first trimester and is complete by 18-20 weeks of gestation; however, the exact gestational age when the invasion of arteries concludes is uncertain. In PE, cytotrophoblast cells infiltrate the decidual part of the placenta instead of the myometrial area of the spiral arteries. The inability of the spiral arteries to develop into wide, convoluted arterial channels caused by the replacement of the musculoelastic wall with fibrinoid material leads to placental hypoperfusion and moderately hypoxic trophoblast tissue, fetal mortality beyond 20 weeks of gestation, abruptio placentae, PE with or without intrauterine growth restriction, intrauterine growth restriction without maternal hypertension, preterm labor, and pre-labor rupture of membranes are all potential complications of a deep placentation abnormality [30].

Defective trophoblast differentiation

Improper trophoblast invasion of the spiral arteries has been linked to a defect in trophoblast differentiation. At the time of endothelial differentiation, which occurs during trophoblast development, the expression of several distinct types of molecules changes [31,32]. The HLA-G molecule is part of the major histocompatibility complex class Ib, along with cytokines, adhesion molecules, extracellular matrix molecules, metalloproteinases, and other molecules. Invading trophoblasts undergo pseudo-vasculogenesis when their adhesion molecule expression changes during normal differentiation from epithelial cell-specific molecules like integrin alpha 6/beta 1, integrin alpha v/beta 5, and E-cadherin to endothelial cell-specific molecules like integrin alpha 1/beta 1, integrin alpha v/beta 3, and VE-cadherin.

Placental hypoperfusion and ischemia

The following evidence points to a connection between placental hypoperfusion, aberrant placental development, and PE. Abnormal placentation and PE are greatly increased in the presence of vascular insufficiency disorders like hypertension, diabetes, systemic lupus erythematosus, renal disease, and thrombophilias [33]. Relative ischemia may occur in situations when there is placental mass but no compromise in placental blood flow, such as in hydrops fetalis, hydatidiform mole, diabetes mellitus, or numerous pregnancies [33]. There may be a correlation between increased incidence and altitude (>3100 meters) in certain circumstances. Caused by abnormal placental development, hypoperfusion may be life-threatening. Due to the inability of the defective uterine vasculature to support the expected rise in blood flow to the baby and placenta, hypoperfusion worsens as gestation progresses. Late placental changes related to ischemia include atherosclerosis, fibrinoid necrosis, thrombosis, sclerotic constriction of arterioles, and placental infarction [34].

Decidual pathology

Failed decidualization may lead to downregulated cytotrophoblast invasion, a phenomenon that was studied. Microarray studies of chorionic villus samples have also revealed a signature of impaired decidualization. Interestingly, decidual cells from women with PE also overexpress sFLT1, suggesting that inadequate suppression of anti-angiogenic factors during the implantation period may lead to shallow implantation [35].

Immunological factors

The research of immunologic variables as a potential contribution to aberrant placental development [36] was motivated by the observation that previous exposure to paternal and fetal antigens seems to have a protective character against PE. Women who are nulliparous, who switch partners between pregnancies, who have long interpregnancy intervals, who use barrier contraception frequently, or who conceive via intracytoplasmic sperm injection are at increased risk for PE, as they are less likely to be exposed to paternal antigens. According to a meta-analysis, women who conceive with the help of an egg donor are more than twice as likely to have PE as those who conceive with the help of any other kind of assisted reproduction. The meta-analysis also indicated that women who undergo artificial conception are four times more likely to develop PE than those who conceive naturally, lending credence to the idea that immunologic intolerance between the mother and fetus contributes to the development of PE. Immunological alterations comparable to those observed in organ rejection modules are present in preeclamptic women. The human leukocyte antigen (HLA) class I antigens HLA-C, HLA-E, and HLA-G are expressed in an unusually high frequency by the extravillous trophoblast (EVT) cells. In the maternal decidua, the EVT cells are found in close proximity to natural killer (NK) cells expressing a range of receptors (CD94, killer cell immunoglobulin-like receptor (KIR), and immunoglobulin-like transcript (ILT)) known to detect class I molecules. The communication between NK cells and EVT cells has been shown to control placental implantation. Patients with PE tend to have lower levels of regulatory T cells (Tregs) in both the systemic circulation and the placental bed, suggesting that this specialized CD4 T cell subset plays a significant role in safeguarding the fetus by moderating the inflammatory immune response. It is hypothesized that a disagreement between the parents’ genes leads to abnormal placental implantation because of elevated NK cell activity, reduced Tregs, and other mediators of the immune response. Biopsies taken from the pre-eclamptic placental bed have shown an increase in the number of dendritic cells infiltrating the decidual tissue. They seem to have a crucial role in setting off antigen-specific T-cell responses to transplanted antigens. Large increases in dendritic cells at the decidual level have been linked to abnormal implantation and a compromised mother's immune response to fetal antigens [37,38].

Genetic factors

PE may have a hereditary component, and heredity has been linked to other diseases. A primigravida’s chance of suffering PE increases two to fivefold if she has a first-degree relative, such as her mother or sister, who also had the condition during pregnancy. The maternal effects of imprinted genes might be considered. The PE phenotype was not displayed in a study of two sisters with PE when the baby or placenta contained the imprinting paternal homolog rather than a maternal STOX1 missense mutation on chromosome 10q22 [39]. There is a seven-fold increased risk of PE in the current pregnancy if there is a prior diagnosis of PE [40]. Women are more likely to get PE than women without this major history if the previous partner of their spouse had PE during the antenatal period. Additionally, it should be highlighted that women who conceive through a man whose prior relationship resulted in PE have a similar likelihood of experiencing it as if the previous partner’s pregnancy had been normal blood pressure [41].

The genes for both sFlt-1 and Flt-1 are located on chromosome 13. More of these gene products will be made by embryos with an additional copy of chromosome 13 (e.g., trisomy 13). The risk of PE in pregnant women carrying a baby with trisomy 13 is well-known to be much higher than in women carrying babies with any other trisomy or in control of pregnant patients. This also contributes to the elevated risk of PE in these women, since their circulating sFlt-1 to placental growth factor (PlGF) ratio is much higher than average. Genome-wide association studies (GWAS) with huge sample sizes have aided in the identification of a genetic risk variation that has a widespread impact [42].

The locus at 12q is associated with HELLP (hemolysis, elevated liver enzymes, and low platelet) syndrome, but not PE without HELLP syndrome, suggesting that the genetic mechanisms at play in HELLP syndrome are separate from those in PE [43]. One possible mechanism that might lead to HELLP syndrome is a change in the long non-coding RNA located at 12q23. Extravillous trophoblast migration may be influenced by the genes regulated by this long noncoding RNA. PAI-1 4G/5G polymorphism, the angiotensinogen gene variation (T235), and endothelial nitric oxide synthase (eNOS) [44] are all genetic regions that have been linked to PE’s development.

Environmental and maternal susceptibility factors

Environmental and maternal susceptibility factors are as follows: (1) low calcium intake; (2) high body mass index; (3) pregnancies conceived using in vitro fertilization (IVF); (4) inflammation: maternal inflammatory symptoms that seem to be present in healthy pregnancies at term are exacerbated by PE; *Chlamydia pneumoniae*, *Helicobacter pylori*, *Cytomegalovirus*, human immunodeficiency virus (both treated and untreated), malaria, herpes simplex virus type 2, bacterial vaginosis, and antibodies to *Mycoplasma hominis* were associated with PE [45]; (5) increased sensitivity to angiotensin 2; (6) complement activation; (7) pre-existing maternal vascular/metabolic/kidney/autoimmune disease: PE is very common in women who already have one or more of the risk factors for vascular disease, including hypertension, diabetes, chronic kidney disease, and autoimmune disorders. Preeclamptic women may be at an increased risk for CVD later in life owing to endothelial damage. Women with a history of PE have an increased risk of developing chronic kidney disease and hypothyroidism [46].

Discussion of the elements used in this study

Mean Arterial Pressure (MAP)

MAP is the measurement of the average pressure in the arteries throughout the course of a complete cardiac cycle (systole and diastole). Multiple variables influence both cardiac output and systemic vascular resistance, which in turn affects MAP. The cardiac output is calculated by multiplying the rate of heartbeats per minute by the volume of each heartbeat. Stroke volume is determined by a variety of factors, including ventricular inotropy and preload. Preload is affected by both blood volume and vein compliance. Boosting cardiac output and stroke volume requires increasing blood volume, which increases preload. As afterload increases, the stroke volume decreases. Heart rate is affected by the myocardium’s chronotropy, dromotropy, and lusitropy.

The following formula is often used to get a MAP estimate: MAP = SBP + 1/3 (diastolic pressure - systolic pressure) DBP minus SBP equals pulse pressure (PP) [47]. The rapid MAP calculation it allows makes it preferable for use in most clinical contexts. MAP ensures the continued viability of all of the body’s tissues by supplying them with oxygen-rich blood. Mechanisms exist to keep the MAP at a steady 60 mmHg, ensuring that blood flows freely to all organs and muscles.

MAP and pregnancy: The chance of developing PE was strongly correlated with the mother’s mean MAP during the first trimester, even after controlling for other potential risk variables. Second-trimester MAP does not reliably predict who will and who will not get the illness.

Pregnancy-Associated Plasma Protein-A (PAPP-A)

Despite its presence in human prenatal plasma, the function of the antigen PAPP-A has remained unclear since its discovery in 1972. Embryonic trophoblast cells secrete a big, highly glycosylated protein termed PAPP-A. Syncytiotrophoblasts produce PAPP-A, a zinc-containing metalloproteinase that binds insulin-like growth factor (IGF), according to the locations of precipitate lines in immunodiffusion experiments [48]. Three more (non-proteolytic) pregnancy-associated proteins were discovered and given the acronyms PAPP-B, -C, and -D. Two disulfide-bound subunits make up the PAPP-A homotetramer, as was discovered. Until the pro-form of eosinophil major basic protein (proMBP) was discovered in 1993, PAPP-A was assumed to be a homotetramer composed of two PAPP-A subunits and two proMBP subunits. The fraction of a pregnant woman’s blood that contains proteins that are not complexed with proMBP but instead circulate as disulfide-bound homodimers is very low (1%). There are possible critical functions for the IGF system in placental growth and development. Therefore, a higher risk of PE is seen in those with low blood levels of PAPP-A. The maternal serum PAPP-A level is increased in symptomatic PE. Within the first several weeks of pregnancy, PAPP-A levels double in about three days and continue to rise progressively afterward. Cleaving insulin-like growth factor binding proteins (IGFBP), particularly IGFBP-4, is how PAPP-A controls the level of free IGF-2. A big heterotetramer comprised of two subunits of PAPP-A and two subunits of pro-major basic binding protein is produced by the placental cytotrophoblast layer and circulates in the blood. To screen for aneuploidy, PAPP-A levels are only measured once, during the first trimester. Pregnancy-induced PAPP-A is complexed with its natural inhibitor, proform of eosinophil major basic protein; in contrast, free PAPP-A has metalloproteolytic activity (proMBP). The only recognized receptors for human IGF-1 are IGFBPs 4 and 5. The release of bound IGF is triggered by these proteins, and this IGF has been demonstrated to activate macrophages, stimulate chemotaxis, increase low-density lipoprotein absorption by macrophages, and cause the production of inflammatory cytokines. On the other hand, current research suggests that IGF may protect against ischemic heart disease by maintaining endothelial function, increasing plaque stability, and acting as an antioxidant and anti-inflammatory. In any case, it is not yet known whether PAPP-A encourages plaque instability or has stabilizing and healing effects on plaque.

These control insulin growth factors, which affect placental development and trophoblast penetration into the maternal decidua. In the maternal circulatory system, it binds to eosinophil major binding protein, blocking its proteolytic function. Because the multifactorial pathogenesis of different PE phenotypes has not been fully elucidated, prevention and prediction are still not possible, and symptomatic clinical management should be mainly directed to prevent maternal morbidity (e.g., eclampsia) and mortality [48]. PE and other negative pregnancy outcomes are associated with decreased PAPP-A levels in the first trimester of pregnancy, as revealed by Karumanchi et al. [49]. It is no secret that trisomy 21 has a biomarker that has been around for a while. PE is more likely to occur in women whose PAPP-A levels are low, as shown by research by Spencer et al. With a PAPP-A cutoff at the 5th centile of normal (multiple of the median (MoM): 0.415; 95% CI: 2.3-4.8), 15% of individuals were diagnosed with PE [50].

Uterine Artery Doppler

During a normal pregnancy, the spiral arteries of the mother undergo a series of modifications at the hands of invasive cytotrophoblasts. To achieve optimum placental perfusion, the fetomaternal circulation must undergo a remodeling process that enhances flow. However, inadequate remodeling of the spiral arteries during placentation has been associated with PE, intrauterine growth restriction, and other related issues [51]. It is possible to non-invasively evaluate the existence of significant uteroplacental resistance using the uterine artery Doppler ultrasonography method. Predicating PE is aided by the uterine artery Doppler screening program. A high pulsatility index (PI) in the uterine artery implies inadequate placentation, which raises the risk of PE, fetal development restriction, abruption, and stillbirth. An abnormally high PI in the uterine artery is defined as one that is more than the 90th percentile. In a normal pregnancy, a woman’s PI in her uterine artery rises in women of African descent and falls in correlation with the length of the baby’s crown to its rump and the mother’s weight gain. When deciding whether or not a certain measurement is normal, these maternal characteristics should be taken into account.

Measurement of uterine artery PI: The internal cervical os and cervical canal may be easily identified in sagittal slices of the uterus. When the transducer is moved slowly from side to side, the blood flow patterns may be mapped in color to pinpoint the exact location of each uterine artery. These arteries, which provide blood to the cervix and uterus, run vertically along either side at the level of the internal os. Whereas if the sampling gate is set to 2 mm and the insonation angle is maintained below 30 degrees, then pulsed wave Doppler may be used to check the whole blood artery. The mean PI of the left and right arteries should be calculated, and the PI and peak systolic velocity (PSV) of the three identical subsequent waveforms should be compared. A PSV in the uterine artery of 60 cm/s is the bare minimum that should be met. When readings are low, it usually means the wrong vessel was checked. If you are doing risk assessments, you need access to the Fetal Medicine Foundation’s Certificate of Competence in Doppler Ultrasound and the 11-13-week scan. It has been demonstrated in a number of studies that the use of maternal characteristics, feasible and cost-effective biomarkers, and uterine artery Doppler may aid in the early prediction of hypertensive diseases in pregnancy.

## Materials and methods

Study design and population

Study Design

It was a prospective observational study of longitudinal variety.

Study Duration

The study was conducted from December 2020 onwards for a period of 18 months. The study was reviewed and permitted by the Institute’s Ethics Committee (Mahatma Gandhi Institute of Medical Sciences, Sewagram, India; approval number: 4663).

Study Subjects

All pregnant women who came to the outpatient department of the institute for routine antenatal check-ups and those who gave consent for the study were the study subjects. These women agreed to deliver at our institute. A special follow-up card was also given.

Sample Size

The number of women admitted to the hospital for labor care during 2019 was 5261. A total of 513 were diagnosed with hypertensive diseases of pregnancy. A vast majority of these patients were admitted through the outpatient department. At a prevalence rate of 10%, we calculated a sample size of 350 to achieve a sensitivity of 85% with an absolute error of 12.5% at a 95% CI.

Inclusion Criteria

Pregnant women included in the study were those cases who reported to the outpatient department with a gestational age of 11-13 weeks during the enrolment period. Women who gave consent were included. Women who got counseled to come for follow-up until delivery were included.

Exclusion Criteria

Exclusion criteria were the following: any pregnant women who do not give consent; any pregnant women who come before 11 weeks and after 13 weeks; multi-fetal pregnancies; women with chronic hypertension; women with existing renal diseases.

Methodology

All pregnant women who came for antenatal check-ups were enrolled in the study. Written and informed consent was obtained from these women in the language they comprehend. Their detailed history was taken and an examination was done, ultrasound examination with Doppler study of uterine artery was done, and serum PAPP-A was tested between 11 and 13 weeks. The details of each component are described below.

Maternal Characteristics

Patients were asked to complete a questionnaire detailing the mother’s demographics, including her age, marital status, occupation, education qualification, number of children, number of pregnancies, and any history of complications. Maternal body mass index (BMI) was determined using the mother’s measured height and weight.

Blood pressure: Mercury sphygmomanometers were used to record the participants’ blood pressure manually, and they were checked for accuracy before and during the research. Doctors with proper training used the equipment to make the recordings. Adult cuffs ranging in size from 22 to 42 centimeters were employed, with the ladies sitting and their arms propped up at heart level. We used MAP = diastolic 13 + systolic 14 to get the average arterial pressure (MAP) (systolic - diastolic). The difference between the two readings was used to determine the pulse pressure. Blood pressure was taken at four visits, i.e., at 11-13 weeks, 14-24 weeks, 25-36 weeks, and more than 37 weeks. MAP was computed for each. All patient data are stored in an Excel spreadsheet (Microsoft Corporation, Redmond, WA), where the formula has been placed for ease of use. While Miller et al. (2007) [52] considered a MAP result vital if it was more than 88 mmHg, we considered a value of 90 mmHg or above to be meaningful.

Uterine Artery Pulsatility Index (UtA-PI)

Ultrasounds using Doppler technology were performed transabdominally throughout the first trimester. The cervical canal and internal cervical os were found in a sagittal segment of the uterus taken between weeks 11 and 13. Next, color flow mapping was performed to locate each uterine artery (UtA) along the cervix and uterine side at the level of the internal os by gently rocking the transducer from side to side. Following the detection of each UtA, the whole vessel was scanned using a pulsed-wave Doppler with a sampling rate of 2 mm.

To avoid examining the arcuate artery, we ensured the insonation angle was less than 30 degrees, and the peak systolic velocity was more than 60 centimeters per second. Measurements of PI were taken from three sequentially recorded waveforms of the same shape, and the PI mean for the left and right arteries was determined. Radiologists with appropriate credentials from the local medical board performed all Doppler tests. Ranges greater than or equal to 1.69 are considered statistically significant for this study’s purposes [53].

Pregnancy-Associated Plasma Protein-A (PAPP-A)

A sample of venous blood (about 3-4 ml) was drawn into a vacutainer and placed in a test tube devoid of the anticoagulant. After the clot formed, the serum was centrifuged and frozen at 80°C for further examination. In this case, chemiluminescence immunoassay equipment was employed. PAPP-A was considered when its value was lower than 0.77. When measured between 11 and 13 weeks post conception, an average PAPP-A level is between 0.77 and 12.6 mIU/ml [54].

Diagnosis of gestational hypertension and preeclampsia

For the most part, we relied on the most recent recommendations by the Working Group on High Blood Pressure in Pregnancy of the National High Blood Pressure Education Program (NHBPEP) [55]. Gestational hypertension is said to be when the SBP is more than or equal to 140 mmHg and DBP is more or equal to 90 mmHg. In a previously normotensive woman, two readings were taken four hours apart without the presence of urine proteins. According to these standards, PE is diagnosed when blood pressure is raised or equal to 140/0 mmHg, with proteinuria or end-organ damage.

Proteinuria is diagnosed when two separate urine samples taken four hours apart show a protein content in the urine of at least 30 milligrams per deciliter (mg/dL) or a reading of 1 on a urine dipstick. Proteinuria was frequently measured using urine dipsticks.

A thorough data sheet was compiled after the MAP, ultrasound results, and women’s characteristics were put into a computer database, including the women’s demographic information. Statistical analysis was done using descriptive and inferential statistics using Pearson’s chi-square test and receiver operating characteristic (ROC) curve and the software used in the analysis was SPSS version 27.0 (IBM Corp., Armonk, NY). P < 0.05 was considered as the level of significance.

## Results

Maternal characteristics

Age

**Figure 1:**
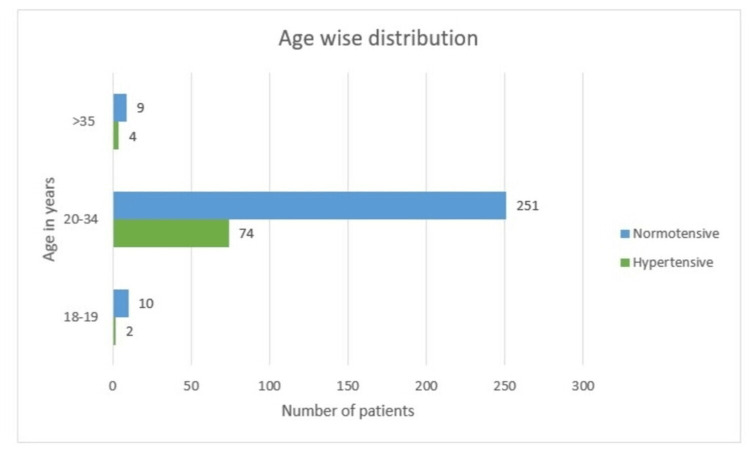
Age-wise distribution

There were 12 (3.4%) females in the teenager group. Of these, two (16.6%) were hypertensive, whereas 10 (83.3%) were non-hypertensive. The maximum number of women (325, 92.9%) falls into the reproductive age group of 20-34 years and the incidence of hypertensive disease of pregnancy was seen in 74 (22.8%) cases. In the elderly age group of >35 years, out of 13 women, four women had hypertensive disease of pregnancy (30.8%), hence hypertension is a significant predictor in elderly aged females, as it is significant at p-value < 0.05 (Table 1 and Figure 1).

**Table 1:** Maternal characteristics

Variables		Total number (%)	Number of hypertensives (%)	Number of non-hypertensives (%)
Age in years	18-19	12 (3.4%)	2 (16.6%)	10 (83.3%)
	20-34	325 (92.9%)	74 (22.8%)	251 (77.2%)
	>35	13 (3.7%)	4 (30.8%)	9 (69.2%)
	Total	350 (100%)	80 (22.9%)	270 (77.1%)
Education	Illiterate	109 (31.1%)	24 (22%)	85 (78%)
	Primary	181 (51.7%)	42 (23.2%)	139 (76.8%)
	Middle	56 (16%)	14 (25%)	42 (75%)
	High	3 (0.9%)	0	3 (100%)
	Graduate	1 (0.3%)	0	1 (100%)
	Total	350 (100%)	80 (22.9%)	270 (77.1%)
Occupation	Housewife	201 (57.4%)	47 (23.4%)	154 76.6%)
	Unskilled worker	148 (42.3%)	33 (22.3%)	115 (77.7%)
	Semi-skilled worker	1 (0.3%)	0	1 (100%)
	Total	350 (100%)	80 (22.9%)	270 (77.1%)
Socioeconomic class				
	Lower-middle	195 (55.7%)	45 (23.1%)	150 (76.9%)
	Lower	155 (44.3%)	35 (22.6%)	120 (77.4%)
	Total	350 (100%)	80 (22.9%)	270 (77.1%)
Gravidity	G1	205 (58.6%)	53 (25.9%)	152 (74.1%)
	G2	105 (30%)	20 (19%)	85 (81%)
	G3	40 (11.4%)	7 (17.5%)	33 (82.5%)
	Total	350 (100%)	80 (22.9%)	270 (77.1%)
Marriage duration	1-3 year	133 (38%)	32 (24.1%)	101 (75.9%)
	4-6 year	194 (55.4%)	45 (23.2%)	149 (76.8%)
	7-10 year	21 (6%)	2 (9.5%)	19 (90.5%)
	11-12 year	2 (0.6%)	1 (50%)	1 (50%)
	Total	350 (100%)	80 (22.9%)	270 (77.1%)
BMI	Normal	244 (69.7%)	57 (23.4%)	187 (76.6%)
	Underweight	98 (28%)	23 (23.5%)	75 (76.5%)
	Overweight	8 (2.3%)	0	8 (100%)
	Total	350 (100%)	80 (22.9%)	270 (77.1%)

Education

**Figure 2:**
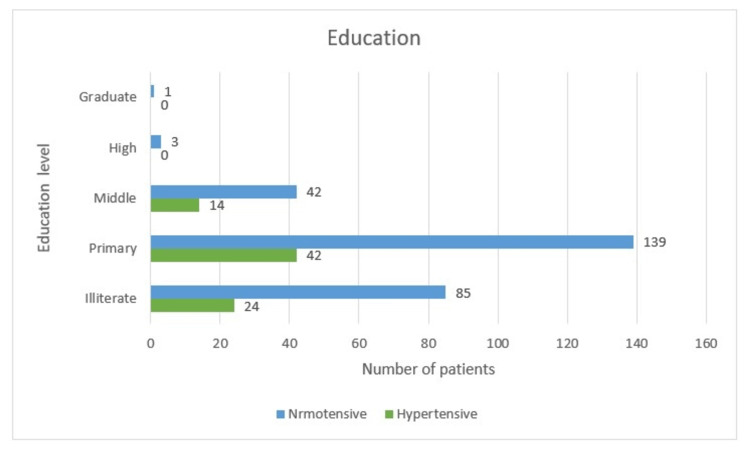
Education-wise distribution

Here, women with primary education dominate the group with 181 people, which comes at 51.7%. This group also hosts 42 (23.2%) of the hypertensives with 139 (76.8%) of the normotensive herd. Leading up second are the illiterate women with 109 (31.1%) cases, with 24 (22%) females who succumbed to hypertension with 85 (78%) normotensive women. A total of 56 (16%) women had attended up to middle school, and among them, 14 (25%) were hypertensive and 42 (75%) were of normal blood pressure. Few women who had completed higher education (3, 0.9%) and a single graduate woman did not suffer from hypertension. On evaluating illiterate, primary, middle, high school, and graduate women who developed hypertension, the results came as between 22% and 25%, which was statistically non-significant as the p-value was <0.05 (Table 1 and Figure 2).

Occupation

**Figure 3:**
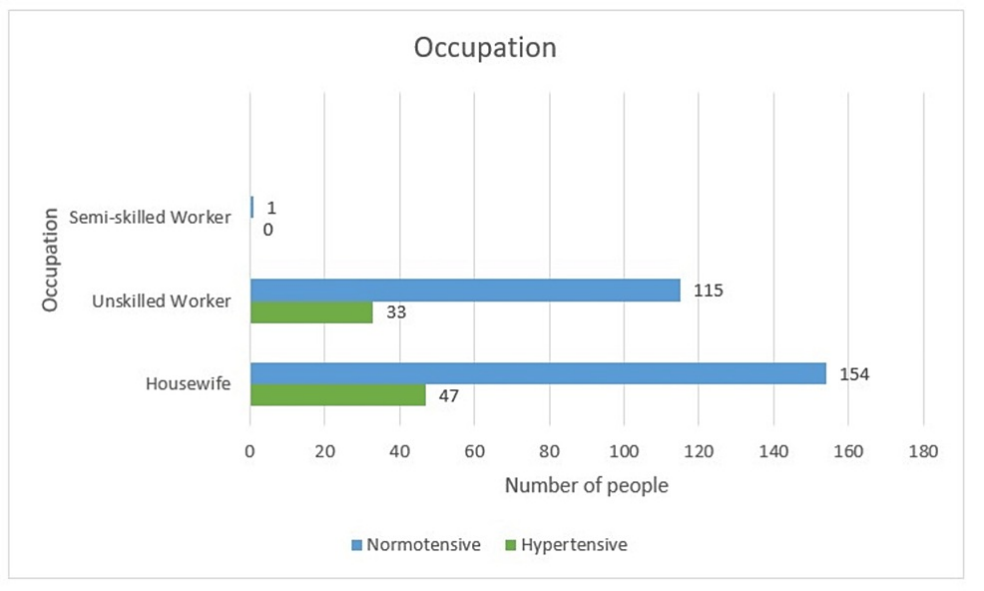
Occupation-wise distribution

The housewives, unskilled workers, and semi-skilled workers constitute 201 (57.4%), 148 (42.3%), and one (0.3%), respectively. Where 47 (23.4%) housewives ailed from hypertension and 33 (22.3%) unskilled workers suffered from the same fate. No cases of hypertension were observed in the semi-skilled worker group. Meanwhile, 76.6%, i.e., 154 housewives and 115 (77.7%) remained normotensive. Again, occupation does not seem to be associated with hypertension as the percentage of women who developed hypertension was between 22% and 23% in all groups with a p-value of 0.838 (Table 1 and Figure 3).

Socioeconomic Status

**Figure 4:**
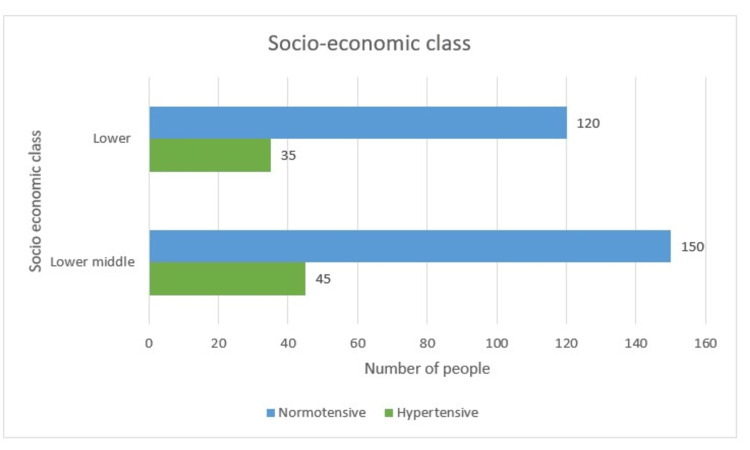
Socioeconomic distribution

The females in this study fall into two categories out of the five in accordance with the modified Kuppuswamy's scale. The lower middle class accounted for the highest numbers, i.e., 195 (55.7%), among which 45 (23.1%) were hypertensive and the rest 150 (76.9%) were normal. A total of 35 (22.6%) out of 155 (44.3%) in the lower socioeconomic group had hypertension while 120 (77.4%) females remained unaffected. No association was found between the development of hypertension and socioeconomic class as the p-value was insignificant at 0.913 (Table 1 and Figure 4).

Gravidity

**Figure 5:**
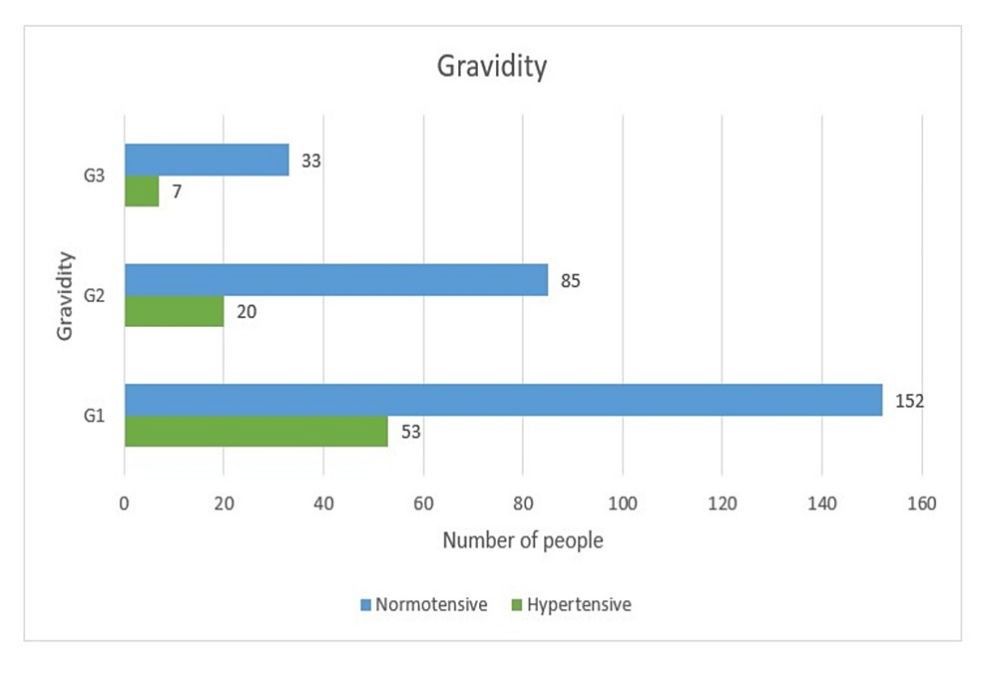
Gravidity-wise distribution

The majority of women (205, 58.6%) were primi gravida, and only 40 (11.4%) were third gravida. Primi gravida had 53 (25.9%) cases who became hypertensive compared to 20 (19%) and seven (17.5%) second and third in multigravida. Therefore, gravidity remains an important maternal characteristic for predicting hypertensive diseases of pregnancy as the p-value remains significant (Table 1 and Figure 5).

Marriage Duration

**Figure 6:**
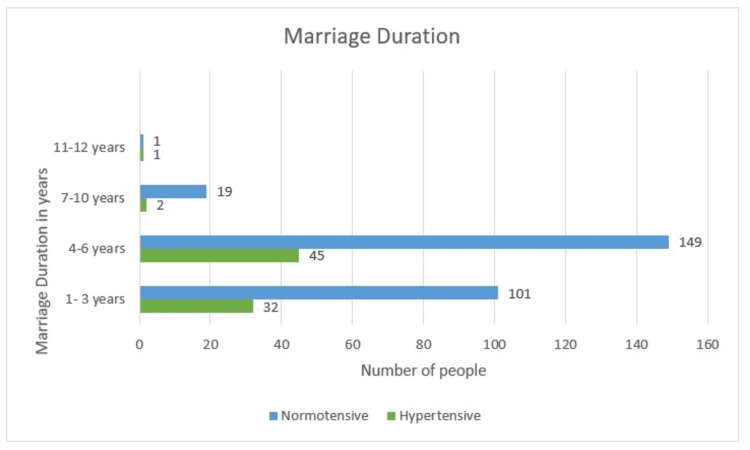
Marriage duration-wise distribution

The duration of four to six years harbors the highest population among others with 194 (55.4%) women. While, one to three years, seven to 10 years, and 11-12 years constituted 133 (38%), 21 (6%), and two (0.6%) of the women. A total of 149 (76.8%) are normotensive in the four to six years duration group. While 101 (75.9%) and 19 (90.5%) have failed to develop hypertension in one to three years and seven to 10 years duration groups, respectively. There were 32 (24.1%) females who had developed hypertension in the one to three years group and 45 (23.2%) in the four to six years group. As the p-value is 0.380, there is no association between hypertension and duration of marriage (Table 1 and Figure 6).

Body Mass Index

**Figure 7:**
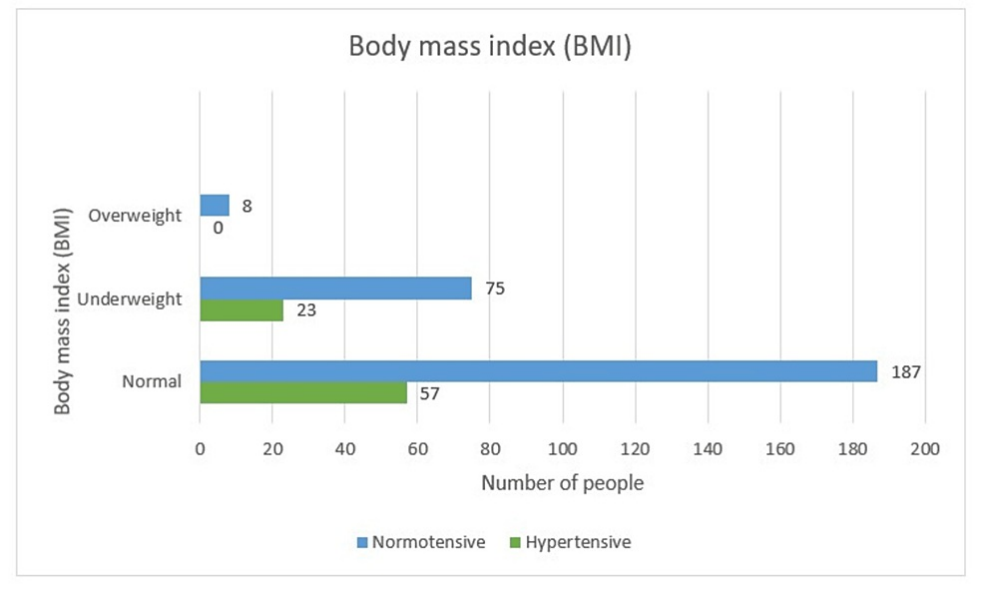
BMI-wise distribution

A total of 57 (23.4%) people developed hypertension in the normal range BMI group, which consisted of 244 (69.7%) females, and 187 (76.6%) remained normotensive. Out of 98 (28%) underweight females, 23 (23.5%) developed hypertension and 75 (76.5%) were normotensive. All eight overweight women surprisingly were normotensive in this study. Hence, BMI in this study was not a predictor for developing hypertensive disease of pregnancy as the p-value is 0.297, i.e., insignificant (Table 1 and Figure 7).

Mean Arterial Pressure

**Figure 8:**
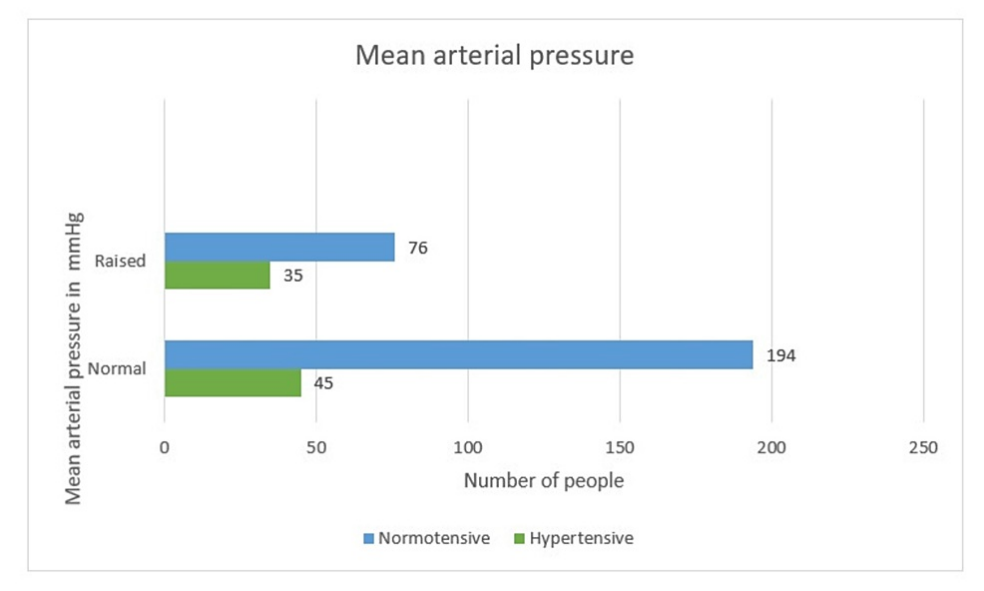
Mean arterial pressure-wise distribution

The MAP was normal in a total of 239 (68.2%) females out of which 45 (18.7%) were hypertensive and 194 (81.3%) were normotensive. While MAP was raised in 111 (31.8%) women, 35 (31.5%) had hypertensive disease of pregnancy. So, MAP is one of the important maternal characteristics that can predict hypertension, as the p-value is less than 0.05 (0.008). Hence, we do have enough proof for association between MAP and hypertension (Table 2 and Figure 8).

**Table 2:** Mean arterial pressure

	Hypertensive	Non-hypertensive	Total
Normal	45 (18.7%)	194 (81.3%)	239 (68.2%)
Raised	35 (31.5%)	76 (68.5%)	111 (31.8%)
Total	80 (22.8%)	270 (77.2%)	350

Biochemical marker - pregnancy-associated plasma protein-A (PAPP-A)

PAPP-A emerged to be on the lower side in 66 (74.1%) cases of hypertension and 23 (25.9%) normotensive cases, which accounts for a total of 89 (25.6%). Whereas, PAPP-A was on the normal side in 14 (5.4%) hypertensive patients and 247 (94.6%) non-hypertensive females, bringing it to a total of 261 (74.6%) cases. Since the p-value for Pearson chi-square is less than 0.05 (0.000), there is an association between PAPP-A and hypertension.

**Figure 9:**
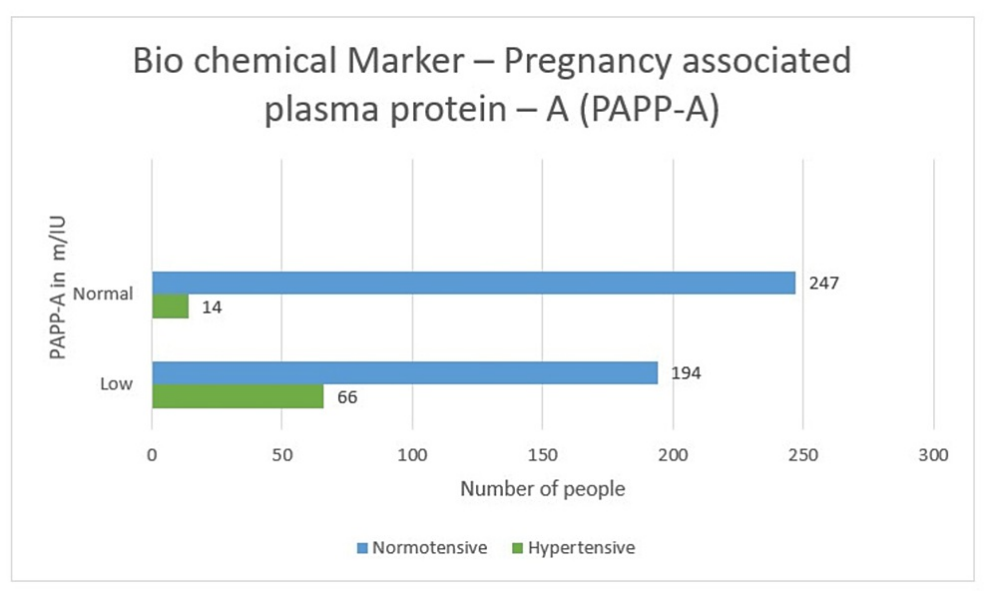
Pregnancy-associated plasma protein-A-wise distribution (graphical)

It was also observed that out of 89 women, where PAPP-A was on the lower side, 66 (74.1%) were hypertensive, so the study clearly states that low serum PAPP-A values are the predictors for hypertensive diseases of pregnancy (Table 3 and Figure 9).

**Table 3:** Pregnancy-associated plasma protein-A-wise distribution

	Hypertensive	Non-hypertensive	Total
Low	66 (74.1%)	23 (25.9%)	89 (25.6%)
Normal	14 (5.4%)	247 (94.6%)	261 (74.6%)
Total	80 (22.8%)	270 (77.2%)	350

Biophysical marker - uterine artery pulsatility index

**Figure 10:**
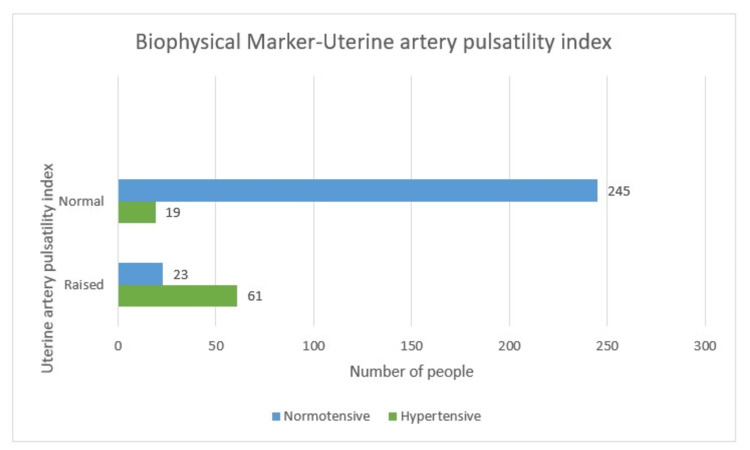
Uterine artery pulsatility index-wise distribution (graphical)

Out of the total positives of 77 (22%) cases, the UtA-PI was raised in 61 (17.4%) hypertensive cases and 16 (4.6%) non-hypertensive cases. There were 19 (5.4%) hypertensive and 254 normotensive (72.5%) cases in a total of 273 (78%). There were 61 (17.4%) hypertensive patients for the positive group, which is significant. Since the p-value for Pearson chi-square is less than 0.05 (0.000), there is an association between UtA-PI and hypertension (Table 4 and Figure 10).

**Table 4:** Uterine artery pulsatility index-wise distribution (tabular)

	Hypertensive	Non-hypertensive	Total
Positive (raised)	61 (17.4%)	16 (4.6%)	77 (22%)
Normal	19 (5.4%)	254 (72.5%)	273 (78%)
Total	80 (22.8%)	270 (77.2%)	350 (100%)

Analysis of the efficiency of the combined screening method

ROC Curve of Mean Arterial Pressure, PAPP-A, and UtA-PI

**Figure 11:**
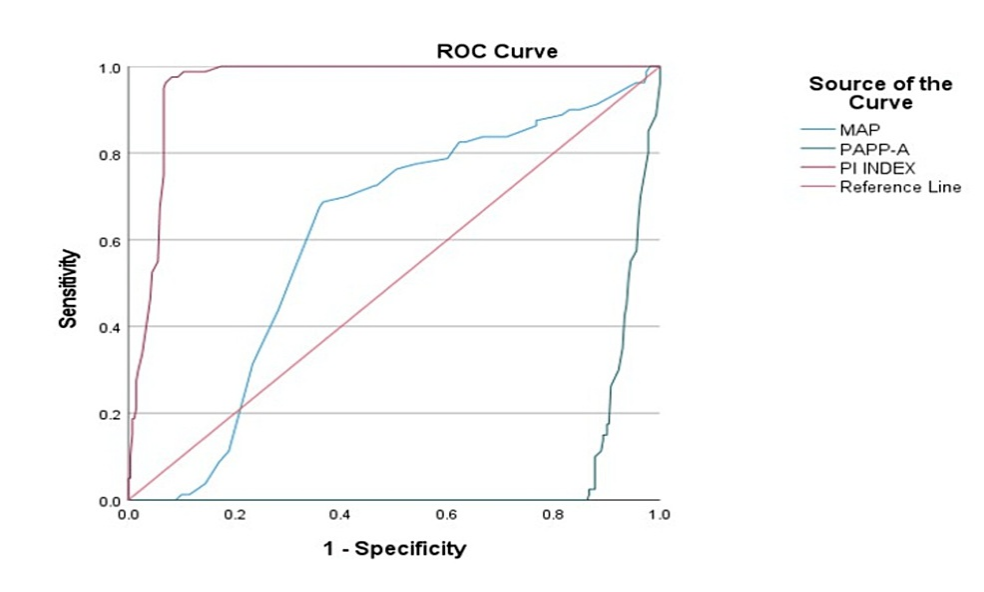
ROC curve ROC: receiver operating characteristic; MAP: mean arterial pressure; PAPP-A: pregnancy-associated plasma protein-A; PI: pulsatility index.

The greater the area under the ROC curve of a particular variable, the better the classifier. Here, our aim is to find the best classifier for predicting hypertension. As we can see from the graph, the PI (UtA-PI) curve has the greatest area, then MAP, and then PAPP-A. Hence, using this graph, we can say that PI is the best classifier that can be used to classify hypertension. After running the logistic regression model, taking hypertension as a dependent variable and MAP, PI-Index, and PAPP-A as independent variables. By looking at the p-value, we found that all three variables are significant and can be used to predict hypertension (Figure 11 and Table 5). Hypertension = (-.10308) * MAP + (-3.70385) * PAPP-A + (6.13524) * PI.

**Table 5:** ROC curve ROC: receiver operating characteristic; MAP: mean arterial pressure; PAPP-A: pregnancy-associated plasma protein-A; UtA-PI: uterine artery pulsatility index.

Variables	Area
MAP	0.608
PAPP-A	0.060
UtA-PI	0.958

Rate of Predictability by Using Combined Methods (Maternal Characteristic, PAPP-A, and UtA-PI)

When all three parameters, i.e., maternal characteristic - MAP, biophysical profile - UtA-PI, and biochemical marker - PAPP-A, were positive, they were able to successfully predict 51 (14.6%) cases accurately, with 12 (3.4%) cases of false positive, 29 (8.2%) cases of false negative, and 258 (73.71%) of true negative cases. The sensitivity comes out to be 63.7%, specificity of 95.5%, positive likelihood ratio of 14.34, negative likelihood ratio of 0.38, disease prevalence of 22.86%, positive predictive value of 80.95%, and negative predictive value of 89.90%, with an accuracy of 88.29% (Table 6).

**Table 6:** Rate of predictability by using combined methods (MAP, PAPP-A, uterine artery PI) MAP: mean arterial pressure; PAPP-A: pregnancy-associated plasma protein-A; PI: pulsatility index.

	Hypertensive	Non-hypertensive	Total
Predicted	51 (14.6%)	12 (3.4%)	63 (18)
Non-predicted	29 (8.2%)	258 (73.71%)	287 (82)
Total	80 (22.8)	270 (77.2)	350

Blood pressure analysis

*Comparison of Systolic Blood Pressure in Normotensive Population During* *Four Visits*

**Figure 12:**
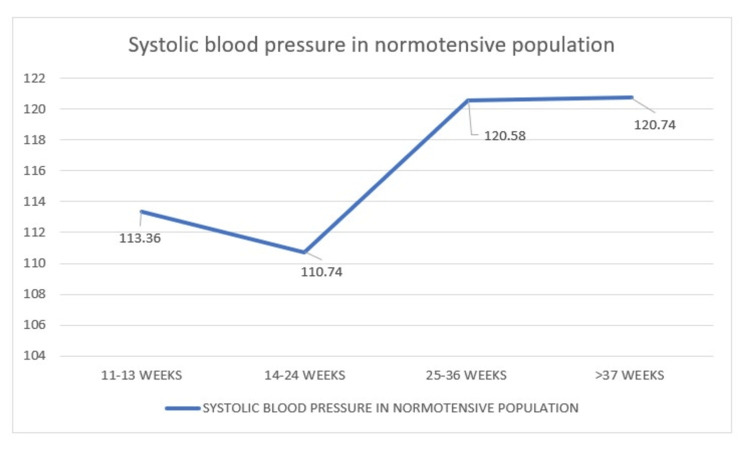
Distribution of systolic blood pressure in normotensive women

Here, there is a comparison of the mean SBP of 270 normotensive women who had visited during 11-13 weeks, 14-24 weeks, 25-36 weeks, and more than 37 weeks for whom the mean pressures were 113.3 mmHg, 110.74 mmHg, 120.58 mmHg, and 120.74 mmHg, respectively. The graph here shows a slight dip at 14-24 weeks followed by almost a plateau after 25 weeks (Figure 12).

Comparison of Diastolic Blood Pressure in Normotensive Population During Four Visits

**Figure 13:**
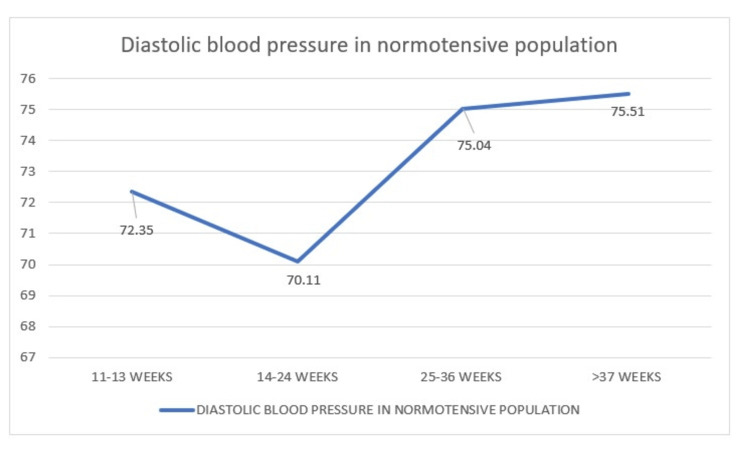
Distribution of diastolic blood pressure in normotensive women

In this study, we have compared the mean DBP of 270 normotensive women who have visited during 11-13 weeks, 14-24 weeks, 25-36 weeks, and more than 37 weeks for whom the mean pressures are 72.35 mmHg, 70.11 mmHg, 75.04 mmHg, and 75.51 mmHg, respectively. The graph here slowly steeps low at 14-24 weeks at 70.11 mmHg followed by an abrupt rise thereafter (Figure 13).

*Comparison of Systolic Blood Pressure in the Hypertensive Population During* *Four Visits*

**Figure 14:**
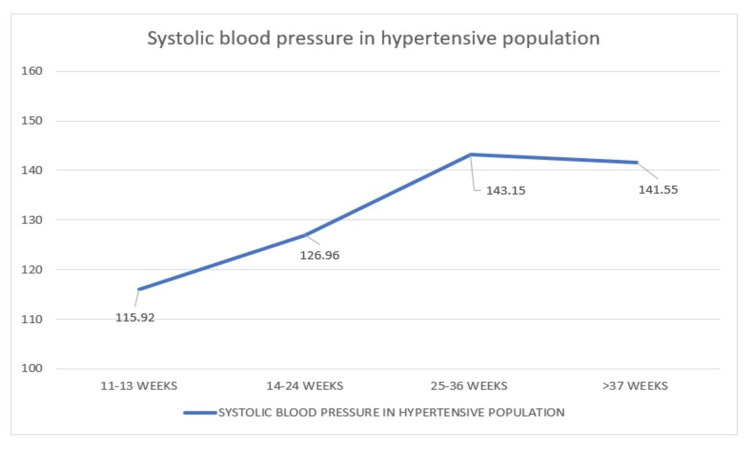
Distribution of systolic blood pressure in hypertensive women

Again, there is a comparison of the mean SBP of 80 hypertensive women who visited during 11-13 weeks, 14-24 weeks, 25-36 weeks, and more than 37 weeks for whom the mean pressures are 115.92 mmHg, 126.96 mmHg, 143.15 mmHg, and 141.55 mmHg, respectively. The graph here swiftly rises followed by a slight downfall after 25 weeks (Figure 14).

*Comparison of Diastolic Blood Pressure in the Hypertensive Population During*​​​​​​​* Four Visits*

**Figure 15:**
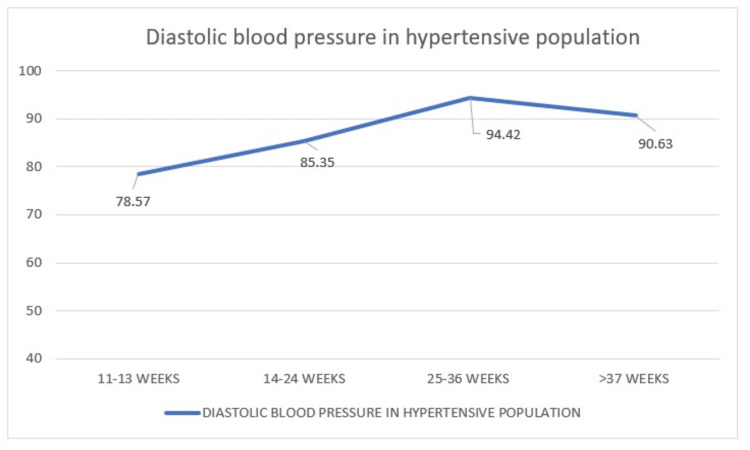
Distribution of diastolic blood pressure in hypertensive women

In this study, we compared the mean DBP of 80 hypertensive women who visited during 11-13 weeks, 14-24 weeks, 25-36 weeks, and more than 37 weeks for whom the mean pressures are 78.57 mmHg, 85.35 mmHg, 94.42 mmHg, and 90.63 mmHg, respectively. The graph here swiftly rises followed by a gradual collapse after 25 weeks (Figure 15).

The main finding of these results is that the women who do not get the mid-trimester fall in blood pressure are the ones who have to be monitored for developing hypertension.

Body edema in relation to hypertensive disease of pregnancy

**Figure 16:**
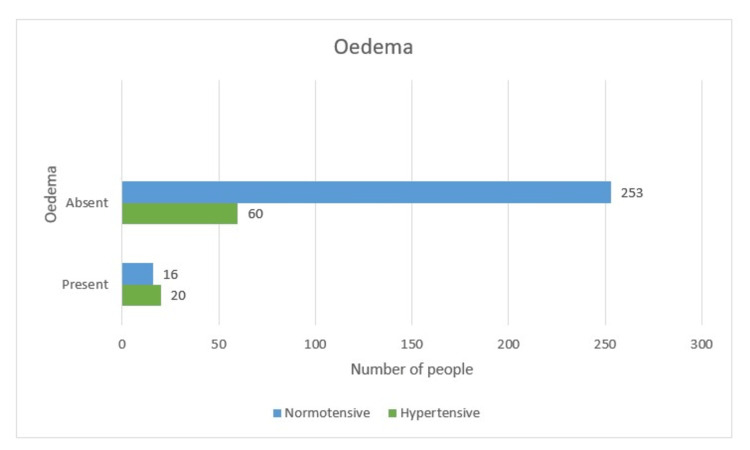
Body edema-wise distribution (graphical)

In the present study, it was observed that 10.6% of cases had edema feet; however, a very interesting finding was that out of 37 cases who had body edema, especially anterior wall edema, 20 (54%) developed hypertension. Whereas out of 313 women who had absent edema, 19.1% had hypertension. This is significant as the p-value is 0.001 (Table 7 and Figure 16).

**Table 7:** Body edema-wise distribution (tabular)

	Hypertensive	Non-hypertensive	Total	P-value
Present	20 (5.7%)	17 (4.9%)	37 (10.6%)	0.001
Absent	60 (17.1%)	253 (72.3%)	313 (89.4%)	
Total	80 (22.8%)	270 (77.2%)	350	

Urine protein

**Figure 17:**
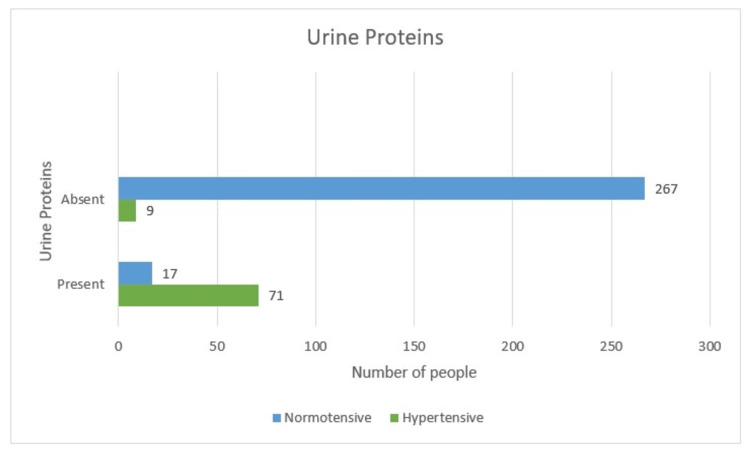
Urine protein-wise distribution (graphical)

Urine protein is found to be present in 71 (20.3%) of hypertensives and three (0.8%) in normotensive females bringing to a total of 74 (21.1%). Out of 276 (78.9%) women, where urine proteins were absent in nine (2.5%) hypertensive and 267 (76.4%) normotensive women. The p-value for Pearson chi-square is less than 0.05 (0.006), hence the presence of urine proteins is strongly associated with hypertension (Table 8 and Figure 17).

**Table 8:** Urine protein-wise distribution (tabular)

	Hypertensive	Non-hypertensive	Total	P-value
Present	71 (20.3%)	3 (0.8%)	74 (21.1%)	0.006
Absent	9 (2.5%)	267 (76.4%)	276 (78.9%)	
Total	80 (22.8%)	270 (77.2%)	350	

Mode of delivery

There were a total of four (1.1%) preterm deliveries of which all were pre-term cesarean sections. Three (0.8%) were from the hypertensive group while one (0.3%) was of the normotensive group. No vaginal deliveries were conducted for the preterm group. In contrast, a total of 322 (92%) term vaginal deliveries took place, where 66 (18.9%) were hypertensive females while 256 (73.1%) were of normotensive nature. Only 24 (6.8%) of total term deliveries were by means of cesarean section. A total of 13 (3.7%) were of normotensive and the rest 11 (3.1%) were of the diseased group. The overall conclusion was that the cesarean section rate was higher in the hypertensive group (17.5%) compared to 5.2% in the normotensive group, which is statistically significant.

Admission to the neonatal intensive care unit

All four preterm babies (1.1%) were admitted to the NICU, of which three (0.8%) had hypertensive mothers while one had a normotensive mother. There were two (0.5%) hypertensive and three (0.8%) normotensive mothers whose babies had to be admitted to the NICU. While all 341 (97.4%) babies were handed to their 75 (21.4%) hypertensive and 266 (76%) normotensive mothers. It was clear from the study that NICU admission in the hypertensive group was 6.25% as compared to the non-hypertensive group, where it was 1.48%. This is statistically significant.

## Discussion

HDP is defined as raised SBP of more than or equal to 140 mmHg systolic and more than or equal to 90 mmHg DBP, recorded after 20 weeks of gestation, two readings, four hours apart in a previously normotensive woman [20,21]. It is one of the causes of maternal mortality in India and it also affects neonatal outcomes [56]. Even with the advent of technology, taking manual blood pressure is the gold standard for diagnosing HDP. Efforts were made by researchers to develop a comprehensive model to predict this calamity as early as 20 weeks to minimize the disastrous maternal complications, reduce their deaths, and subsequently improve neonatal outcomes. Only a few of the models were developed, including the Indian population. Hence, to bridge this gap, an effort has been made through this study. Therefore, this study was performed within a rural tertiary care hospital to develop a comprehensible model using the maternal characteristics - MAP, maternal biophysical profile using UtA-PI, and the biochemical marker - PAPP-A. The observations and their discussion are presented below.

Maternal characteristics

Age

In this study, there were 12 (3.4%) females in the teenager group, of whom 16.6% were hypertensive. The maximum number of women (325) in the reproductive age group had findings of 22.7% cases of hypertension. Glancing at the elderly age group of more than 35 years, four out of 13 women had hypertension accounting for 30.8%. This value is statistically significant, as it was clear that the elderly age group has more cases of hypertension.

Berhe et al. (2018) [57] conducted a study and were of the opinion that pregnant women who are more or equal to 35 years of age are probable to develop hypertension in pregnancy with an odds ratio of 1.64. There was no demonstrable statistically significant difference, which was seen in the hypertension and young female groups (OR = 2.92; 95% CI = 0.88, 9.70), according to this study.

Also, Montan et al. (2007) [58] have observed that increased maternal age is a standalone factor in the development of pregnancy-induced hypertension. His study design also led to the conclusion that many obstetrical complications are associated with an elderly parturient mother. His study is consistent with our findings of the development of hypertension in the elderly group.

Gortzak-Uzan et al. (2001) [59] and Berenson et al. (1997) [60] found that the findings of the development of hypertension in the teenager group were similar to the results of this study.

Hence our findings agree with the literature for the prevalence of hypertension in the elderly population. All the women in the teenage group were married. But still, we received only 12 females may be due to the reluctance of the unmarried females to report to our tertiary care hospital, which is considered a taboo in our society. They prefer to either abort or deliver secretly. Both authors have found that similar incidences and complications were found in the group of young females and that of the middle-aged group.

Education

The primary education group dominates the group with 181 (51.7%) of the population and also comprises 42 (52.5%) of the hypertensives. This was followed by the illiterate women group with 109 (31.1%) cases, with 24 (22%) females with hypertension. A total of 56 (16%) women had attended up to middle school, and among them, 14 (25%) were hypertensive. Few women had completed higher education (3, 0.9%). On evaluating the education component, women who developed hypertension were between 22% and 25%, which was statistically non-significant, as the p-value was < 0.05.

Harris et al. (2020) [61] attempted to find the inconsistencies incurred in health in relation to the hypertensive diseases of pregnancies. He found that members of the African American and Hispanic communities are behind in seeking medical attention due to racism, low levels of education, and social and cultural differences. He was also of the opinion that due to a low level of education, home-based blood pressure monitoring for low-risk hypertensive females was not possible. He has devised a three-step intervention for tackling these issues and reaching this set of women through creating awareness and understanding of the beliefs of the women and imparting education regarding the subject of hypertension.

A retrospective study done by Silva et al. (2008) [62] observed a positive association between lower education level and the development of hypertension when used against controls for the same age and gravidity. He concluded that women with middle-low education with an odds ratio of 1.52 and 95% CI of 1.02-2.27 and low education with an odds ratio of 1.30 and 95% CI of 0.80-2.12 had a greater substantial risk of gestational hypertension than women with high-level education.

The above study was based in the Netherlands, where the components of history taking for low middle and low education groups consisted of maternal substance abuse such as smoking, using of banned drugs, and alcohol consumption; maternal characteristics like raised body mass index found in the latter half of the educational group. Our study is based on a rural Indian population where females have a primary-level education. Also, illegal substance abuse is not seen in these women. Hence, our study could not find a relationship between a low level of education and the development of hypertension. But it was also noteworthy to know that like in the United States, if our rural women are educated about the complications of hypertension, as simple as to care enough to report events like headaches, can prevent disastrous effects.

Occupation

Housewives, unskilled workers, and semi-skilled workers constitute 201 (57.4%), 148 (42.3%), and one (0.3%), respectively. Where 47 (23.4%) housewives ailed from hypertension along with 33 (22.3%) of unskilled workers. Occupation does not seem to be associated with hypertension, as the percentage of women who developed hypertension was between 22% and 23% in all groups with a p-value of 0.838.

Spadarella et al. (2021) [63] did a systemic meta-analysis using 27 eligible studies and found that employed women have a 5.1% more cumulative risk of developing hypertension than non-employed women. Professional and retail jobs are at high risk. Physical workload and shift work are controversial. Physical workload seems to have a protective effect on hypertension. She was of the opinion that clerical, skilled, and service sector jobs, which also could be comparatively less stressful, do not add up to the development of the disease.

Spracklen et al. (2016) [64] found a substantially lower risk of PE when the mothers spent more than 8.25 hours active per day, including both physical activity at work or home and during relaxation time.

Our study findings are similar to the findings of Nugteren et al. (2012) [65], who established that no work-related risk factors, like standing or walking for a long duration, heavy weight lifting, night shifts, and excessive working hours, or any history of exposure to chemicals are related to the development of HDP.

Reviewing the studies, it is then evident that we could not attribute the development of hypertension to one particular group as our rural women, although the majority are housewives, still do not lead a sedentary lifestyle. As being an agrarian society, our females are farmers and lend a hand there. Hence again contributing to non-significant occupational findings.

Socioeconomic Status

Here, the women fell into two categories out of the five in accordance with the modified Kuppuswamy’s scale. The lower middle class accounted for the highest numbers, i.e., 195 (55.7%), among which 45 (23.1%) were hypertensive. A total of 35 (22.6%) out of 155 (44.3%) lower socioeconomic group had hypertension. No association was found between the development of hypertension and the socio-economic class, as the p-value was insignificant at 0.913.

Ospina et al. (2020) [66] was of the opinion that gestational hypertension was one of the low health inequalities observed among the group of low socioeconomic status In Canada. The middle level was small for gestational age and gestational diabetes. The highest level was substance abuse and smoking.

Our study's finding is further supported by the study done by Lawlor et al. (2005) [67], which established that both childhood and adult socioeconomic conditions have no association with the peril of developing HDP. When fully adjusted, the odds ratio at a 95% confidence interval linking those who were born in labor-intensive social classes to those who were born in non-labor-intensive social classes for PE was 1.10 (0.72 to 1.73), and for gestational hypertension, it was 1.02. Parallel results associating females in labor-intensive with non-labor-intensive social classes stratified during each antenatal period were 1.09 for PE and 0.99 for gestational hypertension.

Thus, compared with other studies, it is seen that our women, who belong to the lower socioeconomic status, depend on the daily wages and are laborers. Although in first-world countries, low socioeconomic status is a cause of hypertension, it is also to be seen that their drug and smoking-related habits are very much prevalent in those groups, hence changing the dynamics of maternal health.

Gravidity

The majority of the women (205, 58.6%) were primi gravida, and only 40 (11.4%) were third gravida. Primi gravida had 53 (25.9%) cases who became hypertensive compared to 20 (19%) and seven (17.5%) in the second and third in multigravida. Therefore, gravidity remains an important maternal characteristic for predicting HDP as the p-value remains significant.

Berhe et al. (2018) [57] were of the opinion that there was no such link between pregnancy-induced hypertension and the number of pregnancies with an odds ratio of 1.37.

Sarwar et al. (2016) [68] stated that a higher incidence of hypertension is seen in the primigravidae age group of more than 20 years. Several studies revealed that primigravidas are more prone to developing HDP; however, contradictory research is also available on sporadic disease.

Marriage Duration

In this study, it is seen that the duration of marriage of four to six years has the highest population of 194 (55.4%) women. While, one to three years, seven to 10 years, and 11-12 years constituted 133 (38%), 21 (6%), and two (0.6%) of the women. A total of 32 (24.1%) females had developed hypertension in the one to three years group, and 45 (23.2%) in the four to six years group. Only one developed hypertension in the 11-12 years group. Overall, there is no association between the duration of marriage and the development of hypertension.

Saravade et al. (2021) [69] found an increased incidence of HDP in elderly primi women who were married for a long duration.

A study done by Robillard et al. (1994) [70] concluded that the time of sexual cohabitation before the commencement of sexual acts was contrariwise related to the incidence of HDP. They theorized that the longer a woman is exposed to paternal genetics, the more easier it is to develop immune tolerance.

Body Mass Index

A total of 57 (23.4%) people developed hypertension in the normal range BMI group, which consisted of 244 (69.7%) females. Out of 98 (28%) underweight females, 23 developed hypertension. All eight overweight women surprisingly were normotensive in this study. Hence, according to our study, BMI was not a predictor for developing HDP, as the p-value was 0.297, i.e., insignificant.

Bicocca et al. (2020) [71] researched that obese women (BMI > 30 kg/m2) have a higher risk of developing early and late-onset hypertensive diseases.

Alves et al. (2020) [72] were of the opinion that maternal obesity was a risk for developing gestational hypertension and gestational diabetes and have a higher incidence of cesarean section births.

Gaillard et al. (2011) [73] found that maternal obesity and morbid obesity are strongly linked with the development of high blood pressure in each trimester, and also amplify the risks of HDP.

In our study, we could not establish this link as the majority of our population fell into the lower range of BMI. This may be attributed to the thin-built, malnourished, and short-stature component of the rural population of females. We did not get cases of BMI more than 30 kg/m2, hence BMI did not prove to be a causative factor.

Maternal characteristics - mean arterial pressure

At par with our study, the MAP is normal in a total of 239 (68.2%) females, out of whom 45 (18.7%) were hypertensive and 194 (81.3%) were normotensive. While MAP was raised in 111 (31.8%) women, 35 (31.5%) had HDP. So, MAP is one of the important maternal characteristics that can predict hypertension as the p-value is less than 0.05 (0.008), hence we do have enough proof for the association between MAP and hypertension.

Reddy et al. (2020) [74] did a retrospective cohort study in Australia and found that the measurement of MAP was substantial to the development of maternal hypertension and an effective screening parameter during pregnancy.

Gasse et al. (2018) [75] conducted a study to approximate the predictive value of first-trimester MAP for the hypertensive disorders of pregnancy. At the end of the study, they were of solid opinion that the measurement of first-trimester MAP strongly corresponded with the development of gestational hypertension and PE.

Poon et al. (2010) [76] established that MAP is the best predictor of systolic and diastolic blood pressure and the best screening performer to predict PE.

Poon et al. (2008) [77] have concluded that raised MAP was linked with a hazard of development of hypertension in pregnancy. They developed a screening model that included measurement of MAP along with maternal characteristics like age, BMI, and ethnicity. They were also of the opinion that even if women did not develop hypertension, those with raised MAP during pregnancy were at risk of developing chronic hypertension in the future.

Hence, in synchronous with other studies, MAP is an important tool for the measurement as well as prediction of HDP.

Biochemical profile - pregnancy-associated plasma protein-A

PAPP-A emerged to be on the lower side in 66 (74.1%) cases of hypertension and 23 (25.9%) normotensive cases, which accounts for a total of 89 (25.6%). Since the p-value for Pearson chi-square is less than 0.05 (0.000), there is an association between PAPP-A and hypertension.

Dascau et al. (2020) [78] aimed to study the prediction of HDP using PAPP-A at 11-14 weeks. He found it to be an effective parameter when used alone and also when used with UtA-PI.

Meloni et al. (2009) [79] conducted a study to establish a predictive value for linking low levels of PAPP-A with hypertension. The research was in favor that small levels of serum PAPP-A (0.8 MoM) may be a latent source for early screening of expectant women as they are at a higher risk of developing HDP. A total of 111 (8.9%) out of 973 pregnant women over three years were found to have hypertension and the result with the help of ROC curve statistics revealed that a PAPP-A MoM value <0.8 was able to significantly predict hypertension in pregnancy with p < 0.001 and the area under the ROC curve of 83%.

Yaron et al. (2008) [80] performed research with respect to the decreased value of PAPP-A. They discovered that when the level of PAPP-A is less than 25 MoM, it is linked to a range of maternal and fetal outcomes, including aneuploidy, non-proteinuric HDP, fetal growth restriction, and spontaneous abortion.

After reviewing other authors and from our findings, PAPP-A measurement is a good predictor of maternal hypertension.

Biophysical profile - uterine artery pulsatility index

Out of the total positives of 77 (22%) cases in this study, UtA-PI was raised in 61 (17.4%) hypertensive cases and 16 (4.6%) non-hypertensive cases. There were 19 (5.4%) hypertensive and 254 normotensive (72.5%) cases of a total of 273 (78%). There were 61 (17.4%) hypertensive patients for the positive group, which is significant. Since the p-value for Pearson chi-square was less than 0.05 (0.000), there is an association between UtA-PI and hypertension.

Shinde et al. (2021) [81] found success in the prediction of hypertension in pregnancy by employing UtA-PI as early as 11 to 13 weeks.

Khong et al. (2015) [51] suggest that first-trimester uterine artery Doppler has much higher predictive accuracy than late-onset PE in exposing early-onset PE and fetal development limitation.

According to Plasencia et al. (2007) [82], maternal characteristics, such as history, ethnicity, and body mass index, as well as measurements of UtA-PI at 11-13 weeks of gestation, are helpful in the early diagnosis of PE.

Utilizing UtA-PI alone or in combination has proved to be of immense important biophysical profile in the early detection of HDP. Some authors solely use this for prediction.

Predictive accuracy of the combined screening method using maternal characteristic (MAP), biophysical profile (UtA-PI), and biochemical profile (PAPP-A)

According to our ROC analysis that used the logistic regression model, taking hypertension as a dependent variable and MAP, UtA-PI, and PAPP-A as independent variables, by looking at the p-value, we found that all three variables are significant and can be used to predict hypertension. Hypertension = (-.10308) * MAP + (-3.70385) * PAPP.A + (6.13524) * PI.

The statistical analysis shows that the sensitivity comes out to be 63.7%, with a specificity of 95.5%, positive likelihood ratio of 14.34, negative likelihood ratio of 0.38, disease prevalence of 22.86%, positive predictive value of 80.95%, and negative predictive value of 89.90% with an accuracy of 88.29%.

Ji-jun et al. (2022) [83] have confirmed that adding PlGF to MAP, UtA-PI, and PAPP-A combination improves the detection of hypertensive disorders of pregnancy. Hu et al. (2021) [84], using a combination of MAP, UtA-PI, and PAPP-A, created a screening technique in China to detect preterm PE with the following results detection rates for preterm PE of 65.0%, 72.7%, and 76.1%, respectively.

Sapantzoglou et al. (2021) [85] found that the addition of cell-free fetal DNA did not improve the already combined screening model using MAP, UtA-PI, and PAPP-A.

Zumaeta et al. (2020) [86] found that a similar detection rate can be accomplished but at a higher screen-positive rate when PAPP-A was used instead of PlGF in the combined screening methods.

According to research conducted in Iran by Masihi et al. (2016) [87], PAPP-A dropped while MAP and UtA-PI increased. PAPP-A fared less than UtA-PI, with a cut-off point of 2.1 having a specificity of 83.7% and a sensitivity of 100% in predicting hypertensive diseases. Predicting hypertensive disorders in the first trimester, particularly early PE, was facilitated with a combination of UtA-PI, MAP, and PAPP-A.

Selvaraj et al. (2016) [88] commented that the predictive efficacy for detecting PE and fetal growth restriction is fairly good when PAPP-A is added to MAP and UtA-PI.

Kumar et al. (2015) [89] revealed that when the maternal characteristic of BMI was added to the MAP, UtA-PI, and PAPP-A, the sensitivity and specificity of the test were 73% and 70%, respectively, and it was a good predictor of early-onset PE.

Scazzocchio et al. (2013) [90] comprised a model of MAP, UtA-PI, and PAPP-A to identify early PE in routine care, low-risk scenario. A total of 5170 people were included, and of those, 136 (2.6%) experienced early PE, while 26 (0.5%) did not. A total of 110 (2.1%) women had late PE. At 5% and 10% false-positive rates, the detection rates for early PE were 69.2% and 80.8% (area under the curve: 0.95; 95% CI: 0.94-0.98), while for late PE, they were 29.4% and 39.6% (area under the curve: 0.71; 95% CI: 0.66-0.76).

Poon et al. (2010) [91] found that the inclusion of PAPP-A, when paired with MAP and UtA-PI at 11-13 weeks, improves the effectiveness of screening for early PE. The detection rate of early PE in screening for a combination of uterine artery left pulsatility index, MAP, and PAPP-A was 83.8%, with a 5% false-positive rate.

After reviewing the literature, it is evident that a combined screening method using maternal characteristics (MAP), biophysical profile (UtA-PI), and biochemical profile (PAPP-A) is an effective model for predicting hypertension as early as 11-13 weeks of gestation. Also, its availability makes it appropriate for usage in rural populations.

Blood pressure changes during pregnancy in normal and hypertensive patients

After reviewing the graphs showing the mean systolic and diastolic blood pressure for both normotensive and hypertensive women at numbers 6-9, it was found that both the systolic and diastolic blood pressure changes trimester-wise in accordance to the physiology as given in the literature.

Hermida et al. (2000) [92] monitored around 1494 patients and our findings were similar to her research. In healthy pregnant women, up to the halfway point of the pregnancy, blood pressure gradually drops; after that, it gradually rises until delivery day. When a woman has gestational hypertension or PE, her blood pressure remains steady during the first half of pregnancy before steadily rising until birth.

Body edema in relation to hypertension

In the present study, it was observed that 10.6% of cases had edema feet; however, of 37 cases who had body edema, especially anterior wall edema, 20 (54%) developed hypertension. This is significant as the p-value is 0.001. The findings are consistent with the physiology and sign of raised blood pressure as the amount of water in the body as a whole rises by 6 to 8 liters, of which 4 to 6 liters are extracellular and at least 2 to 3 liters are interstitial. Between the mother’s extracellular compartments and the product of conception, there is a total sodium retention of roughly 950 mmol. Thus, a slight decrease in interstitial fluid colloid osmotic pressure and an increase in capillary hydrostatic pressure, as well as changes in the hydration of connective tissue ground material, are all associated with variations in local starling forces, according to Davison (1997) [93].

Urine proteins and maternal hypertension

Urine proteins are found to be present in 71 (20.3%) of hypertensives of a total of 74 (21.1%). Out of 276 (78.9%) women, urine proteins were absent in nine (2.5%) hypertensive cases. The p-value for Pearson chi-square is less than 0.05 (0.006), hence the presence of urine proteins is strongly associated with hypertension.

Yıldız et al. (2022) [94] found the levels of protein in urine can be co-related with adverse maternal and neonatal outcomes in hypertensive females.

Serum total protein levels and the degree of proteinuria were noted at PE diagnosis and delivery, according to Morikawa et al. (2020) [95]. It predicted a worse maternal outcome when urine proteins were positive.

Thus detection of urine proteins remains an important factor of association with the prediction and outcome of hypertension in expectant mothers.

Mode of delivery and hypertension

In this study, the cesarean section rate was more in the hypertensive group (17.5%) compared to 5.2% in the normotensive group, which is statistically significant.

Dassah et al. (2019) [96] concluded that there is a higher rate of cesarean section for expectant mothers with HDP in a tertiary care center in Ghana.

Stella et al. (2008) [97] were of the opinion that there is a significant increase in the number of cesarean sections for hypertensive mothers (odds ratio of 1.62 and confidence interval of 1.47-1.78).

Our findings are at par with the findings of Gofton et al. (2001) [98] that there is an increase in obstetrical operative interventions, including cesarean sections in women with hypertension. Hence, it is seen that there is an increase in births by cesarean section in women with hypertension.

Time of delivery and maternal hypertension

In our study, out of 350, only four (1.1%) preterm deliveries had taken place, of which all were preterm cesarean sections. Three (0.8%) were from the hypertensive group.

Adu-Bonsaffoh et al. (2019) [99] commented that there was an increase in pre-term births attributed to maternal hypertension, advanced maternal age of the mother, and premature rupture of membranes.

Sibai (2006) [100] explored PE as a cause of pre-term delivery in patients with hypertension.

These findings are consistent with our study, as all three hypertensive mothers who had to be delivered preterm had emergency cesarean sections due to uncontrolled hypertension despite adequate anti-hypertensive therapy. Out of whom one patient had persistent decreased fetal movements with poor non-stress test.

NICU admission and hypertension

Our study revealed that NICU admission in the hypertensive group was 6.25% as compared to the non-hypertensive group, where it was 1.48%, which is statistically significant.

Abdelazim et al. (2020) [101] have cited that hypertensive mothers, especially those diagnosed with PE, have a higher chance of perinatal morbidity like low Apgar score, low birth weight, pre-term delivery, and subsequent higher NICU admissions.

Similarly, Stella et al. (2008) [97] commented on the increase in NICU admissions due to maternal hypertensive diseases.

When fetal growth restriction, ventilator assistance, and respiratory distress syndrome were investigated in pregnant mothers with hypertensive disease, Hauth et al. (2000) [102] showed that there was an increase in NICU hospitalizations.

It is evident from the above literature and with our findings that the predictive accuracy for the three combined screening factors at 11-13 weeks of pregnancy employing the use of maternal characteristic MAP, biophysical profile UtA-PI, and biochemical profile PAPP-A is far more superior, and to an extent cost-effective for the rural population as simple blood pressure measurement is feasible at every visit and uterine artery Doppler is being performed along the first-trimester scan. Hence, with the following evidence stated, it can be accepted that a new model for screening gestational hypertension has been developed. PAPP-A test is slightly expensive, but it can be subsidized if used in regular screening. However, in comparison to the cost of the treatment of morbidities associated with HDP, this single test when combined with others is very cost-effective. This test not only helps in HDP screening but also gives an idea about genetic malformations, especially in elderly gravidas.

So, this study strongly recommends combined screening for early detection of HDP.

## Conclusions

The discussion revolves around the development and testing of a comprehensive model for predicting hypertensive diseases of pregnancy, a significant cause of maternal mortality in India with implications for neonatal outcomes. The study aims to bridge gaps in existing models, particularly for the rural Indian population. It focuses on maternal characteristics, including age, education, occupation, socio-economic status, gravidity, marriage duration, BMI, as well as biophysical and biochemical profiles. The maternal characteristics analysis reveals that older age, lower education levels, and specific occupations are associated with higher incidences of hypertension. However, socio-economic status does not show a significant correlation. Gravidity and marriage duration are explored, with longer marriage durations not necessarily linked to hypertension. BMI, despite being a known risk factor in other populations, does not prove significant, potentially due to the thin and malnourished nature of the rural population.

The study then delves into specific maternal characteristics: MAP, PAPP-A, and UtA-PI. MAP and PAPP-A are identified as predictors of hypertensive diseases of pregnancy, consistent with existing literature. The UtA-PI also shows a significant association with hypertension, and a combined screening method using these factors is proposed. The model's statistical analysis suggests high sensitivity, specificity, and predictive values, highlighting its potential for early detection. The study further explores blood pressure changes during pregnancy, body edema, urine proteins, and their associations with hypertension. Cesarean section rates, preterm deliveries, and NICU admissions are also analyzed in hypertensive mothers, demonstrating higher rates compared to normotensive counterparts. The discussion concludes by emphasizing the importance of the proposed combined screening method for the early detection of HDP. Despite the slightly higher cost of PAPP-A testing, the study argues that its inclusion in regular screening is cost-effective when compared to the potential costs of treating associated morbidities. The model not only aids in hypertensive disease screening but also provides insights into genetic malformations, particularly in elderly gravidas, making a strong case for its adoption in prenatal care.
